# Supplementary material for: Resolving the full spectrum of human genome variation using Linked-Reads
Source: Genome Res. 2019 Apr;29(4):635–45. doi: 10.1101/gr.234443.118 (PMC6442396; doi:10.1101/gr.234443.118)
Supplement: Supplemental Material [file supp_gr.234443.118_Supplemental_longranger-2.2.zip › longranger-2.2.tar.bz2!/lr2.2-public/lib/stan/cmdstan/stan/doc/api/html/math_2matrix_2trace__gen__quad__form_8hpp_source.html]

Stan: src/stan/math/matrix/trace\_gen\_quad\_form.hpp Source File


|  |  |
| --- | --- |
|  | Stan  2.5.0  probability, sampling & optimization |


- Main Page
- Related Pages
- Namespaces
- Classes
- Files

- File List
- File Members

All Classes Namespaces Files Functions Variables Typedefs Enumerations Enumerator Friends Macros Pages

- src
- stan
- math
- matrix

trace\_gen\_quad\_form.hpp

Go to the documentation of this file.

1 #ifndef STAN\_\_MATH\_\_MATRIX\_\_TRACE\_GEN\_QUAD\_FORM\_HPP

2 #define STAN\_\_MATH\_\_MATRIX\_\_TRACE\_GEN\_QUAD\_FORM\_HPP

3

4 #include <boost/utility/enable\_if.hpp>

5 #include <boost/type\_traits.hpp>

6 #include <stan/math/matrix/Eigen.hpp>

7 #include <stan/math/error\_handling/matrix/check\_multiplicable.hpp>

8 #include <stan/math/error\_handling/matrix/check\_square.hpp>

9

10 namespace stan {

11  namespace math {

15  template<int RD,int CD,int RA,int CA,int RB,int CB>

16  inline double

17  trace\_gen\_quad\_form(const Eigen::Matrix<double,RD,CD> &D,

18  const Eigen::Matrix<double,RA,CA> &A,

19  const Eigen::Matrix<double,RB,CB> &B)

20  {

21  stan::math::check\_square("trace\_gen\_quad\_form(%1%)",A,"A",(double\*)0);

22  stan::math::check\_square("trace\_gen\_quad\_form(%1%)",D,"D",(double\*)0);

23  stan::math::check\_multiplicable("trace\_gen\_quad\_form(%1%)",A,"A",

24  B,"B",(double\*)0);

25  stan::math::check\_multiplicable("trace\_gen\_quad\_form(%1%)",B,"B",

26  D,"D",(double\*)0);

27  return (D\*B.transpose()\*A\*B).trace();

28  }

29  }

30 }

31

32 #endif

33

check\_multiplicable.hpp

stan::math::check\_multiplicable

bool check\_multiplicable(const char \*function, const T1 &y1, const char \*name1, const T2 &y2, const char \*name2, T\_result \*result)

**Definition:** check\_multiplicable.hpp:15

Eigen.hpp

stan::math::trace

T trace(const Eigen::Matrix< T, Eigen::Dynamic, Eigen::Dynamic > &m)

Returns the trace of the specified matrix.

**Definition:** trace.hpp:20

stan::math::trace\_gen\_quad\_form

double trace\_gen\_quad\_form(const Eigen::Matrix< double, RD, CD > &D, const Eigen::Matrix< double, RA, CA > &A, const Eigen::Matrix< double, RB, CB > &B)

Compute trace(D B^T A B).

**Definition:** trace\_gen\_quad\_form.hpp:17

stan::math::check\_square

bool check\_square(const char \*function, const Eigen::Matrix< T\_y, Eigen::Dynamic, Eigen::Dynamic > &y, const char \*name, T\_result \*result)

Return true if the specified matrix is square.

**Definition:** check\_square.hpp:24

check\_square.hpp


---

[ Stan Home Page ]

*© 2011–2014,
Stan Development Team.*
